# KD-409, a Respiratory Syncytial Virus FG Chimeric Protein without the CX3C Chemokine Motif, Is an Efficient Respiratory Syncytial Virus Vaccine Preparation for Passive and Active Immunization in Mice

**DOI:** 10.3390/vaccines12070753

**Published:** 2024-07-08

**Authors:** Ryo Yamaue, Masaharu Torikai, Madoka Terashima, Hiroaki Mori

**Affiliations:** KM Biologics Co., Ltd., Kikuchi Research Center, 1314-1 Kyokushi Kawabe, Kikuchi-shi 869-1298, Japan; yamaue-ryo@kmbiologics.com (R.Y.); terashima-ma@kmbiologics.com (M.T.);

**Keywords:** RSV vaccine, FG chimeric protein, central conserved domain, CX3C chemokine motif, passive immunity, minimum effective dose

## Abstract

Although respiratory syncytial virus (RSV) vaccine development initiatives have existed for half a century, no candidate has been approved for application at all ages from neonates to children. Developing an effective and safe RSV vaccine for pediatric use is challenging owing to RSV-associated disease and vaccine-enhanced disease (VED). We aimed to design an RSV vaccine, KD-409, by structurally incorporating the F ectodomain and G protein central conserved domain without the CX3C chemokine motif and test its efficacy and safety. KD-409 formed rosette particles or trimmers. KD-409 immunization of mice mainly induced anti-RSV F protein IgG. The induced anti-F antibodies had a higher IgG2a/IgG1 ratio than pre-fusion F, suggesting that they induced Th1-dominant immunity. Active and passive immunities were assessed by analyzing the viral titers in BALB/c mice intranasally challenged with RSV after intramuscular KD-409 immunization and pups derived from mothers who were intramuscularly vaccinated with KD-409 twice, respectively. KD-409 was more effective than post-fusion F and had a lower minimum effective dose than pre-fusion F. Thus, KD-409 demonstrated great potential as a novel RSV vaccine candidate, outperforming existing RSV F-based candidates. Our findings provide a promising strategy to overcome RSV-associated acute lower respiratory infections without the risk of VED associated with traditional approaches.

## 1. Introduction

Almost every human has been infected with respiratory syncytial virus (RSV) at least once before reaching the age of 2 years [1,2]. Neonates and older adults are likely to experience more severe symptoms of RSV infection. In 2019, 33.0 million RSV-associated acute lower respiratory infection (RSV-ALRI) episodes, 3.6 million RSV-ALRI hospital admissions, 26,300 RSV-ALRI in-hospital deaths, and 101,400 RSV-ALRI total deaths were estimated in children aged 0–60 months worldwide [3]. In infants aged 0–6 months, 6.6 million RSV-ALRI episodes, 1.4 million RSV-ALRI hospital admissions, 13,300 RSV-ALRI in-hospital deaths, and 45,700 RSV-attributable deaths were estimated to have occurred [3]. On 3 May 2023, the Food and Drug Administration (FDA) approved the world’s first RSV vaccine, AREXVY from GSK [4], for adults aged 60 years and older. Although Pfizer has conducted a Phase 3 clinical trial in pregnant women and newborns (NCT04424316), the results have not accomplished all primary endpoints [5,6]; however, the FDA approved Pfizer’s maternal RSV vaccine, named ABRYSVO, to protect newborns on 21 August 2023 [7]. The use of ABRYSVO in the elderly has also been approved [8]. AREXVY and ABRYSVO are not intended for use in children, and there is no licensed pediatric RSV vaccine. Current vaccine strategies tend to comprise passive immunization for infants and active immunization for children and adults.

RSV F and G proteins are major antigens that have shown promise for developing an RSV vaccine. These proteins are localized on the surface of viral envelopes and involved in RSV infection of host cells. RSV surface F proteins deliver the genome into the host cell by fusing the viral envelope to the host cell membrane, transitioning from pre-fusion F (pre-F) to post-fusion F (post-F) in response to infection [9,10]. In previous studies, the sera from mice immunized with pre-F showed a higher neutralizing antibody titer than those from mice immunized with post-F. Immunization with pre-F leads to the production of RSV F site Φ-specific antibodies, such as D25, which have a significantly higher neutralization potency than antibodies targeting RSV F sites I, II, III, and IV [11,12]. Alternatively, RSV G functions as an attachment protein for host cells. The RSV G protein sequence is more variable between viral strains than the RSV F protein sequence; however, there is a highly conserved region, the central conserved domain (CCD), which contains a CX3C motif that interacts with CX3CR1 on the cell surface to promote infection [13,14]. Monoclonal antibodies against CCD, such as 3D3, 131-2G, 2B11, and 3G12, have a neutralizing capacity comparable to that of anti-RSV F antibodies [15,16]. In particular, when using human airway epithelial cells with retained CX3CR1 as primary cultured cells, a neutralizing ability of anti-G antibodies has been observed [13]. In addition to the CX3C motif, the CCD contains a heparin-binding domain (HBD), the function of which is to attach to heparan sulfate on the cell surface [17].

As mentioned earlier, immunization with RSV F or G has been shown to induce the neutralizing activity of anti-RSV F or G protein antibodies. However, despite this promising aspect, there are several challenges associated with the use of RSV F or G protein as vaccine antigens. As a vaccine candidate, RSV post-F has not demonstrated sufficient efficacy. Additionally, there are concerns regarding the stability of the protein structure and the potential for exacerbating inflammation in lung tissues after RSV challenge following immunization with an RSV pre-F vaccine [18,19]. Clinical trials involving formalin-inactivated RSV (FI-RSV) have reported instances of post-vaccination exacerbation of symptoms, known as vaccine-enhanced disease (VED) [20]. VED was associated with vaccination in young, presumably RSV-naïve children and has not been considered a risk in older children or adults. Furthermore, a clinical trial of BBG2Na, which includes residues of RSV A G_130–230_ (G2Na) fused to the albumin-binding domain of streptococcal G protein (BB), had to be discontinued owing to rare adverse events [21]. RSV G-protein-derived side effects have been linked to the CX3C motif, suggesting that its interaction with CX3CR1 is responsible for exacerbating symptoms [22,23]. Some of these side effects have been better understood, but a definitive cure or vaccine for children is still lacking.

Interestingly, in a BALB/c mouse model, administering anti-RSV G antibodies significantly reduced airway hyper-responsiveness 7 days after RSV infection compared to anti-RSV F antibody administration [24]. This finding indicates that antibodies targeting specific regions of the G protein (3G12 and 3D3) at G_167–176_ and G_164–172_, respectively, which do not include the problematic CX3C motif of the CCD, may offer both effectiveness and safety [24].

While RSV F-protein-based vaccines have been the predominant focus of development, there is a growing interest in exploring the potential of vaccines centered around the G protein near the CCD. In this study, we aimed to develop a novel chimeric RSV vaccine, KD-409, harboring both the F and G antigens of RSV. KD-409 included the highly conserved region of the RSV G protein without the CX3C motif. We also evaluated the efficacy of KD-409 in initiating passive and active immunity in mouse models in vivo. KD-409 demonstrated remarkable potential as a novel RSV vaccine candidate, outperforming existing RSV F-based candidates. These findings can be valuable in overcoming RSV-associated acute lower respiratory infections without the risk of VED associated with traditional approaches.

## 2. Materials and Methods

### 2.1. Recombinant Protein Expression and Purification

Plasmid DNA was introduced into 7.5 × 10^7^ Expi293F cells (A14527, Thermo Fisher Scientific K.K., Tokyo, Japan) and cultured at 37 °C, 8% CO_2_, 125 rpm for 4 days with agitation to transiently express various recombinant proteins (wild-type [WT] F, post-F, pre-F, FG). F protein sequences were derived from those of RSV A strains. The post-F sequence was derived from the 1–513 sequence from SEQ ID NO: 1 listed in MedImmun’s patent WO2016144675A1. Pre-F contains DS-Cav1 mutations, including S155C, S290C, S190F, and V207L. The FG chimera has a portion of the FP domain of the F protein replaced by DFHFEVFNFV and 10 amino acids derived from the G protein sequence. Site IV glycosylation of FG was introduced through mutations K419N, K421T, T434N, and S436T in the F protein sequence. WT F, post-F, and FG chimera have 6 x His-FLAG tags, while pre-F has a trimerization tag, Foldon, and 6 x His-Strep tag at the C-terminus. The culture supernatant was centrifuged, filtered, and purified using Ni-NTA Agarose (QIAGEN K.K., Tokyo, Japan). These recombinant proteins were also obtained through Dulbecco’s phosphate-buffered saline (D-PBS; FUJIFILM Wako Pure Chemical Corporation, Osaka, Japan) displacement and filtration through a 0.22 μm filter. Details regarding “Recombinant Protein Expression” are described in patent application WO2020175660 [25]. For the minimum drug dose evaluation only, non-tagged KD-409 was derived from CHO DG44 cells (Meiji Seika Pharma Co., Ltd., Tokyo, Japan) cultured and purified using three-step chromatography. The FI-RSV was prepared based on the Lot 100 formulation manufactured by Pfizer, Inc. in the mid-1960s, as previously described [20,26]

### 2.2. Virus Preparation

HEp-2 cells (CCL-23; ATCC, Manassas, VA, USA) were seeded into 225 cm^2^ flasks at 1 × 10^5^ cells/mL and pre-cultured at 37 °C with 5% CO_2_. The cells were cultured with RSV A2 (VR-1540; ATCC) in Minimum Essential Medium (MEM; Thermo Fisher Scientific K.K.) supplemented with 2% fetal bovine serum (FBS; Global Life Sciences Technologies Japan K.K., Tokyo, Japan) at 1–4.5 × 10^5^ cells/mL for 3 days at 37 °C and 5% CO_2_. After centrifugation (390× *g*, 5 min, 4 °C), the supernatant was collected, and 225 cm^2^ flasks were washed with D-PBS. The cells were detached using 0.25% Trypsin/EDTA (Thermo Fisher Scientific K.K.) and collected in MEM supplemented with 2% FBS. After centrifugation (390× *g*, 5 min, 4 °C), the supernatant was removed, and the cell pellet was suspended in 4 mL of 25% sucrose/PBS. The cell suspension was frozen in liquid nitrogen, thawed, pipetted 50–60 times, and centrifuged (390× *g*, 5 min, 4 °C), after which the supernatant was collected.

### 2.3. Analysis of Physical Properties: Dynamic Light Scattering

The particle sizes of various proteins were measured using Zetasizer Nano (Malvern Panalytical Ltd., Grovewood Road, UK) as per the manufacturer’s instructions.

### 2.4. Analysis of Physical Properties: Size-Exclusion Chromatography

Particle size was measured using gel filtration chromatography with the Agilent 1200 Series system (Agilent Technologies, Inc., Santa Clara, CA, USA) and Superdex 200 Increase 5/150 GL (Global Life Sciences Technologies Japan K.K., Tokyo, Japan). The samples were filtered through a 0.22 μm filter after dilution with D-PBS. Gel Filtration Standard (Bio-Rad Laboratories, Inc., Hercules, CA, USA) was used as the molecular weight standard.

### 2.5. Analysis of Physical Properties: Electron Microscopic Observation

Recombinant protein samples were processed via negative staining with saturated uranium acetate and 2% phosphotungstic acid, fixed overnight in 4% glutaraldehyde, and then observed with an electron microscope (TecnaiG2 12 TWIN; FEI Company, Hillsboro, OR, USA).

### 2.6. Electrophoresis

Samples (1 µg) were heated at 98 °C for 5 min and loaded onto a Bolt™ Bis-Tris Plus Mini Protein 4–12% gel (Thermo Fisher Scientific K.K.). Electrophoresis was performed at 200 V for 30 min. The gel was stained with Bullet CBB Stain One (Nacalai Tesque, Inc., Kyoto, Japan) for 1 h, washed, and photographed using WSE-6100 Lumino Graph I (Atto Corporation, Tokyo, Japan).

### 2.7. Anti-RSV F Antibody Titer

Recombinant RSV pre-F protein diluted in D-PBS was applied to 96-well Pierce Nickel Coated Plates (Thermo Fisher Scientific K.K.) and allowed to stand at 2–8 °C overnight or 37 °C for 1 h. After washing the plates with PBS, they were blocked with 1% bovine serum albumin (BSA). Mouse serum was then applied, and the plates were incubated at 37 °C for 1 h. After washing with PBS-Tween (PBST), an anti-mouse IgG horseradish peroxidase (HRP)-conjugated antibody (Global Life Sciences Technologies Japan K.K.) diluted in 1% BSA was applied, and the plates were incubated at 37 °C for 1 h. Subsequently, the plates were washed with PBST, and 3,3’,5,5’-tetramethylbenzidine liquid substrate (Sigma-Aldrich Co. LLC, St. Louis, MO, USA) was added, followed by incubation at 25 °C for 30 min. Results were measured using SPECTRAMAX 190 (Thermo Fisher Scientific K.K.).

### 2.8. Anti-RSV G Antibody Titer

Following the manufacturer’s recommendations, G_158–172_ peptides modified with N-terminal carboxyl residues (Eurofins Genomics K.K., Tokyo, Japan) were immobilized on Pierce Maleimide Activated Plates (Thermo Fisher Scientific K.K.), washed with PBST, and blocked with 1% BSA; excess maleimide groups were inactivated. The pretreated plates were reacted with mouse immune serum, HRP-Rabbit Anti-Mouse IgG (H + L; Thermo Fisher Scientific K.K.) was added, and the plates were made fluorogenic using the QuantaRed Enhanced Chemifluorescent HRP Substrate Kit (Thermo Fisher Scientific K.K.). Measurements were performed using Synergy HT (BioTek Instruments, Winooski, VT, USA). Details regarding the above protocol can be found on the manufacturer’s website [27,28].

### 2.9. Neutralizing Antibody Titer

HEp-2 cells (CCL-23; ATCC) were seeded in 96-well plates at 2 × 10^5^ cells/mL and pre-cultured at 37 °C and 5% CO_2_ for 1 day. Serum and RSV diluent containing rabbit serum complement (Cedarlane, Ontario, Canada) were mixed in equal volumes and incubated at 37 °C and 5% CO_2_ for 1 h. After removing the culture supernatant from the plate, the serum–RSV reaction solution was added and incubated at 37 °C and 5% CO_2_ for 3–5 days. Then, after removal of the reaction solution and washing with PBS, methanol was added, and the plates were allowed to stand at 25 °C for 30 min. Following the removal of methanol, air-drying, and washing with PBS, the anti-RSV F antibody diluent was applied and incubated at 37 °C for 1 h. Next, after removal of the anti-RSV F antibody diluent, the anti-mouse IgG Alexa488-conjugated antibody (Abcam, Cambridge, UK) diluent was applied and incubated at 37 °C for 1 h. The cells were washed with PBS and diluted Hoechst 33342 solution (DOJINDO LABORATORIES, Kumamoto, Japan) was applied, followed by incubation in the dark for 10 min. The cells were analyzed using an image analyzer (Image Xpress Micro XLS; Molecular Devices, LLC., San Jose, CA, USA). The infection rate of the wells to which only RSV was applied and that of the serum dilution series were used as references. The analysis was performed using an image analyzer (Image Xpress Micro XLS; Molecular Devices). The analysis method was as follows: the relationship between the serum dilution factor and the infection inhibition rate was plotted, and the infection rate of the wells to which only RSV was applied was considered to be 100%. Neutralizing antibody titer (IC_50_) was calculated through curve fitting using GraphPad Prism 9 (GraphPad Software Inc., San Diego, CA, USA).

### 2.10. Anti-RSV F Antibody Subclass Analysis

Basic methods are described in Section 2.7. The HRP-labeled antibodies used in this study were Goat Anti-Mouse IgG1 (HRP) pre-adsorbed (ab98693, Abcam) and Goat Anti-Mouse IgG2a (HRP) pre-adsorbed (ab98698, Abcam), both of which specifically targeted mouse IgG. Immune sera were evaluated from 30 pooled sera samples.

### 2.11. Viral Copy Number Measurement

#### 2.11.1. Alveolar Lavage Fluid

After centrifugation (300× *g*, 3 min, 25 °C) of bronchoalveolar lavage fluid (BALF) and supernatant collection, viral RNA was isolated from 150 μL of supernatant with NucleoSpin RNA Virus (MACHEREY-NAGEL, Nordrhein-Westfalen, Denmark). The High-Capacity cDNA Reverse Transcription Kit (Applied Biosystems, Waltham, MA, USA) was used to synthesize cDNA from the extracted RNA. The protocol was performed according to the manufacturer’s recommendations.

#### 2.11.2. Lung Tissues

Lung tissues were collected in Lysing Matrix D (MP-Bio Japan K.K., Tokyo, Japan) tubes, and TRIzol (Thermo Fisher Scientific K.K.) was added. The tubes were agitated with FastPrep-24 (MP-Bio Japan K.K.), and the lung tissue was crushed. cDNA synthesis was performed from the extracted RNA using the High-Capacity cDNA Reverse Transcription Kit (Thermo Fisher Scientific K.K.) following the manufacturer’s instructions.

Quantitative polymerase chain reaction (PCR) was performed to calculate the viral copy number, with standard DNA used as calibration curve in the range of 10^2^–10^7^ copies. A mixture of the sense primer (CARCAAAGTTAYTCTATCATGTC), an antisense primer (GATCCTGCATTRTCACARTACCA), a minor groove binder (MGB) probe (TGTAGTACAATTRCCACT; the standard DNA was modified with a labeled fluorescent dye on the 3’ side, and the 3’ side was modified with the MGB and Eclipse Quencher [MGBEQ]), and distilled water (NIPPON GENE CO., LTD., Tokyo, Japan) was used to prepare the pre-treatment for the samples, as described above. Standard DNA (TGTCCAACAATGTTCAAATAGTTAGACAGCAAAGTTACTCTATCATGTCCATAATAAAAGAGGAAGTCTTAGCATATGTAGTACAATTACCACTATATGGTGTTATAGATACACCCTGTTGGAAACTACACACATCCCCTCTATGTACAACCAACACAAAAGAAGGGTCCAACATCTGTTTAACAAGAACTGACAGAGGATGGTACTGTGACAATGCAGGATCAGTATCTTTCTTCCCACAAGCTGAAACATGTA) was used to obtain 10^2^–10^7^ copies. Real-time PCR was performed 50 times by cycling at 50 °C for 2 min, followed by 95 °C for 10 min, 95 °C for 30 s, and 60 °C for 1 min. A calibration curve was prepared from the amplification curve of the standard, and the virus copy numbers of the samples were calculated using QuantStudio 7 (Applied Biosystems). Samples below the detection limit were analyzed at the lower limit of 100 copies.

### 2.12. Animal Experiments: Immunogenicity

Animals: Female BALB/c mice, aged 5–6 weeks (Japan SLC, Inc., Shizuoka, Japan), were acclimated for about 1 week before use (n = 32 for neutralizing titer, n = 30 for IgG2a/IgG1 ratio, and n = 16 for anti-F and anti-G antibody titer). Immunization: Various antigens were prepared at a dose of 5 μg and administered intramuscularly twice at 3-week intervals (50 μL per leg). In the adjuvant groups, Adju-Phos (InvivoGen, San Diego, CA, USA) was used at a dose of 6 μg per animal. Collection: Three weeks after the second immunization, total blood samples were collected under 2–3% isoflurane anesthesia, and serum was obtained after centrifugation (3000× *g*, 10 min, 15–25 °C).

### 2.13. Animal Experiments: Active Immunization Model

Animal and immunization details are the same as those described in the section titled “Animal Experiments: Immunogenicity” (n = 207 for BALF RSV A2 viral load). Challenge: Three weeks after the second dose of various antigens, RSV A2 at 1 × 10^5^ plaque forming units (PFU) was inoculated intranasally (20 μL in one nostril) under 2–3% isoflurane anesthesia. Collection: Mice were euthanized with carbon dioxide gas 3 days after virus inoculation. BALF samples were obtained by washing and collecting twice with 0.5 mL of D-PBS (FUJIFILM Wako Pure Chemical Corporation, Osaka, Japan).

### 2.14. Animal Experiments: Passive Immunization Model

Animals: Female BALB/c mice aged 5–6 weeks (Japan SLC, Inc.) and male BALB/c mice aged over 8 weeks (Japan SLC, Inc.) were acclimated for about 1 week before use (n = 128 for Lung RSV A2 viral load). Immunization: Various antigens were prepared at a dose of 15 μg and 0.5 ng administered twice at 3-week intervals (intramuscular injection; 50 μL per leg; the first dose was administered at 5–7 weeks of age). Adju-Phos (InvivoGen) was used at a dose of 6 μg per animal. Mating: Two females per male were placed in a cage, starting 2 weeks after the first immunization, and the mating period was 1 week before the second dose. Challenge: RSV A2 at 1 × 10^5^ PFU was inoculated intranasally (10 μL in one nostril) into approximately 2-week-old (14–19 days) pups under 2–3% isoflurane anesthesia. Collection: Pups were euthanized with carbon dioxide gas 3–4 days after virus inoculation and whole lungs were collected.

### 2.15. Ethics Statement

The study protocols were approved by the KM Biologics Institutional Animal Care and Use Committee.

### 2.16. Statistical Analysis

Neutralizing antibody titers and viral loads were summarized with geometric means and 95% confidence intervals (CIs). Antibody subclass analyses were summarized using the arithmetic mean and 95% CI. Analyses were performed with the available values, and no missing data were imputed. For neutralizing antibody titer and antibody subclass analysis, the two-tailed Mann–Whitney U test was performed as a statistical test. In the Animal Experiments Active Immunization Model, the two-tailed Mann–Whitney U test or Kruskal–Wallis test and Dunn’s multiple comparison test were used. In the Animal Experiments Passive Immunization Model, the Kruskal–Wallis test and Dunn’s multiple comparison test were performed. Statistical analysis was performed using GraphPad Prism software version 9.5.1 (GraphPad Software Inc.).

## 3. Results

### 3.1. Structure and Characterization of KD-409

The RSV F protein is being developed as a universal vaccine antigen owing to its low variability among virus strains. In contrast, G proteins display significant variability among viral strains, except for the CCD (G_157–198_). The CCD includes a highly conserved region (CCD-high; G_164–176_), a cysteine noose (a characteristic structure formed by disulfide bonds between cysteine residues 173–186 and 176–182), and a CX3C chemokine-like motif (G_182–186_) [29,30]. The RSV G protein sequence 162–171 contains a portion of CCD-high and a linear epitope of the anti-RSV G antibody 3D3 [16]. The G protein CX3C motif not only adheres to host cells but also binds to CX3CR1+ cells and reduces antiviral T cell responses [31,32]. In particular, RSV G_162–171_ is not directly involved in RSV adhesion to host cells and symptom exacerbation but is instead a peripheral region [29].

**Figure 1:**
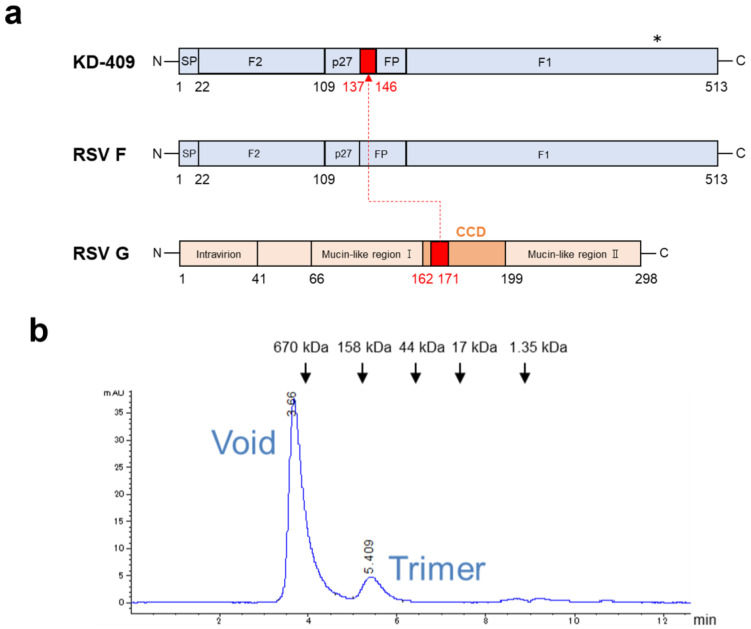

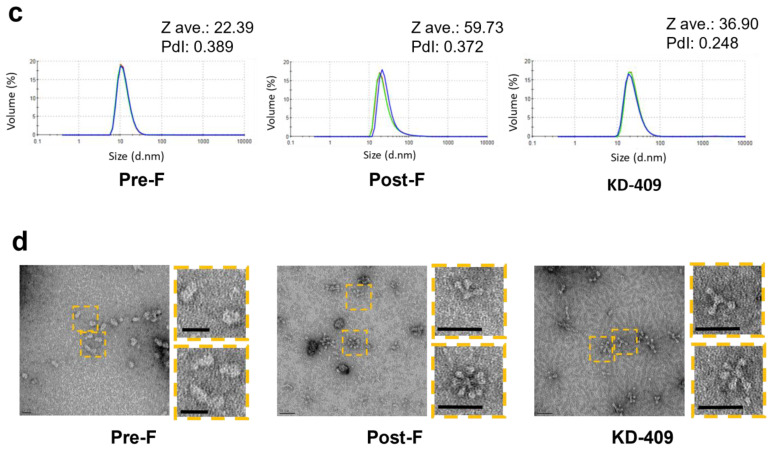
Design of respiratory syncytial virus (RSV) FG chimeric proteins. (**a**) KD-409 is based on the basic structure of RSV F sequence 1–513, except for the transmembrane region, and a portion of the FP domain, RSV F_137–146_, is replaced by RSV G_162–171_. Glycosylation is introduced around site IV, indicated by *. The K419N, K421T, T434N, and S436T mutations introduce N-type or O-type glycosylation. (**b**) Size-exclusion chromatography trace of trimeric and rosette-like particle structures. Positions of molecular weight standards are indicated with arrows. (**c**) Evaluation of the size distribution of KD-409 with dynamic light scattering. (**d**) Evaluation of KD-409, post-fusion F (post-F), and pre-fusion F (pre-F) using transmission electron microscopy. Inserts show zoomed views of each original image. Scale bar, 20 nm (pre-F) and 50 nm (post-F and KD-409).

KD-409 was designed by replacing the RSV F protein FP domain sequence 137–146 with the highly conserved RSV G protein sequence 162–171, harboring the CCD without the CX3C domain (Figure 1a). Site IV is a common epitope on the surface of pre-F and post-F, but KD-409 was artificially glycosylated around site IV to improve protein expression. According to a previous study, glycosylation of site IV increases the expression of this FG chimeric protein by approximately 5.8-fold [25]. The full-length WT F is estimated to be approximately 68 kDa in its monomeric form, and WT F (1–513 aa), which represents the ectodomain, has a molecular weight of approximately 57 kDa, calculated from the amino acid sequence. The bands in the SDS-PAGE of KD-409 were shifted to the polymer side compared to those of pre-F and post-F; the KD-409 bands were separated into F1 and F2 due to reduction, as in pre-F and post-F (Figure S1). Although the calculated molecular weights are nearly identical, the apparent molecular weight of KD-409 is suggested to be increased by artificial glycosylation of site IV. The sizes of pre-F and post-F are reported to be 11 and 17 nm, respectively (PDB IDs 4JHW and 3RRR, respectively) [33]. The characteristics of KD-409 were measured using dynamic light scattering and size-exclusion chromatography. The dynamic light scattering results indicated a Z-average of 36.90 nm, which was larger than pre-F and smaller than post-F. Additionally, the main peak exceeded the detection limit of 670 kDa, while a second peak of approximately 139 kDa was observed in the size-exclusion chromatography results (Figure 1b,c). Electron microscopic images indicated that KD-409 appeared to form a rosette-like structure (Figure 1d), suggesting that KD-409 exists as trimeric and rosette-like particle structures rather than monomers. KD-409 exhibited reactivity with anti-RSV F antibodies targeting epitopes, such as site Φ, site I, site II, site III, and site IV. In comparison to pre-F, KD-409 showed reduced reactivity with anti-site Φ antibodies (Figure S2). These results indicate that KD-409 resembles WT/post-F more than pre-F in the overall structure, including particle formation.

### 3.2. Immunogenicity of RSV FG Chimeric Protein

Immunization with pre-F induces antibodies against the pre-F-specific site Φ, which exhibit a higher neutralizing capacity than antibodies against site II. As a result, pre-F immunization generates higher neutralizing antibody titers in immune sera than post-F immunization [12,34]. F, G, and SH proteins are found on the surface of the RSV envelope, with pre-F and G being particularly crucial factors in the establishment of infection, and antibodies against each protein show a high neutralizing capacity [16,35]. Expecting that having antibodies to both F and G could enhance neutralizing capacity, we analyzed antibody and neutralizing antibody titers in sera from mice immunized with KD-409.

**Figure 2:**
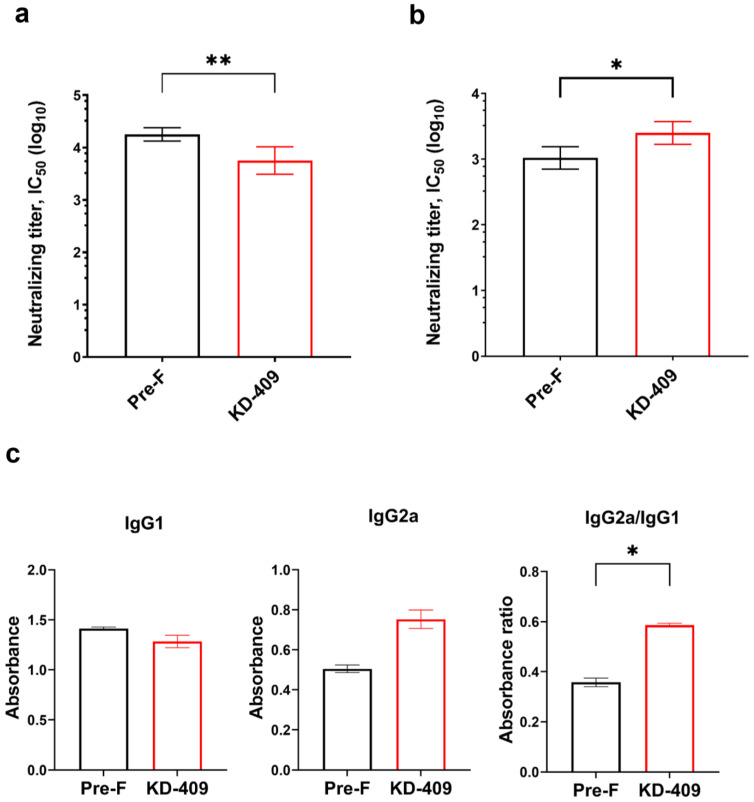
Evaluation of antibodies induced by KD-409. (**a**) Neutralizing antibody titers were measured in the absence of complement. The half-maximal inhibitory concentration (IC_50_) of pre-F and KD-409 immune sera are shown. Error bars indicate the 95% confidence interval (CI). Statistical analysis was performed with the two-tailed Mann–Whitney U test (** *p* < 0.01, n = 8). (**b**) Neutralizing antibody titers were measured in the presence of complement. The IC_50_ of pre-F and KD-409 immune sera are shown. Error bars indicate 95% CI. Statistical analysis was performed with the two-tailed Mann–Whitney U test (* *p* < 0.05, n = 5–8). (**c**) IgG2a/IgG1 ratio of anti-F antibodies in immune sera is shown. Error bars indicate S.D. Statistical analysis was performed with the two-tailed Mann–Whitney U test (* *p* < 0.05, pooled serum from 30 animals).

Anti-G antibodies were not detected in WT F immune sera, while anti-G antibodies were detected in KD-409 immune sera but at lower concentrations than anti-F antibodies. In the serum of mice inoculated twice with KD-409 and aluminum phosphate, the anti-F antibody concentration was 362.2 ± 43.85 µg/mL (mean ± S.D.). In contrast, the anti-G antibody concentration was 3.1 ± 0.4 µg/mL (mean ± S.D.), which is approximately 1/118th of the concentration of the anti-F antibody. Regarding neutralizing antibody titer (IC_50_) in the absence of complement, pre-F immune sera showed higher values than KD-409 immune sera (Figure 2a). However, neutralizing antibody titers (IC_50_) in the presence of complement, which resembles the serum environment, showed markedly higher values for KD-409 immune sera than for pre-F immune sera (Figure 2b). Subclass analysis of anti-F antibodies revealed that KD-409 induced a higher IgG2a/IgG1 ratio than pre-F (Figure 2c), suggesting a Th1-predominant response. These results suggest that KD-409 outperformed pre-F in neutralizing ability in the presence of complement, which concurs with the biological environment.

### 3.3. Protective Efficacy of KD-409 against Infection (Active Immunity)

**Figure 3:**
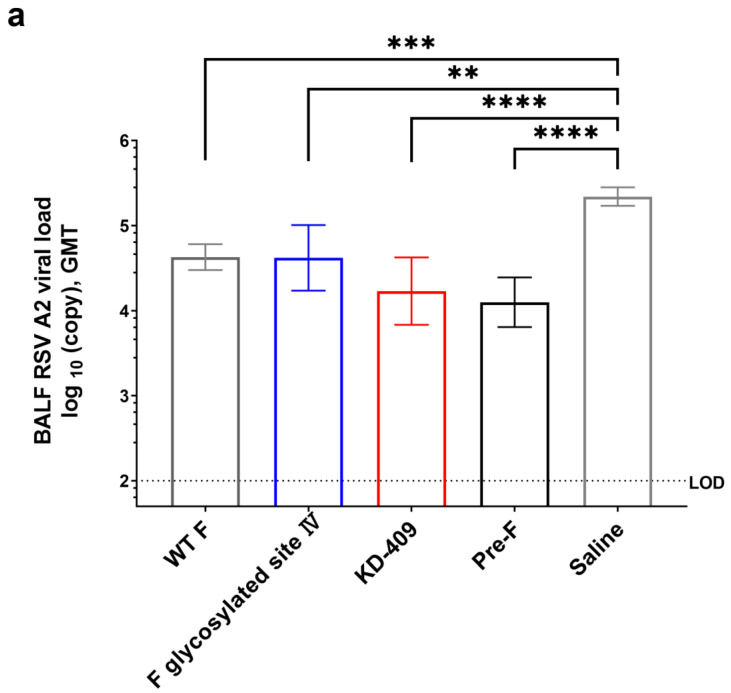

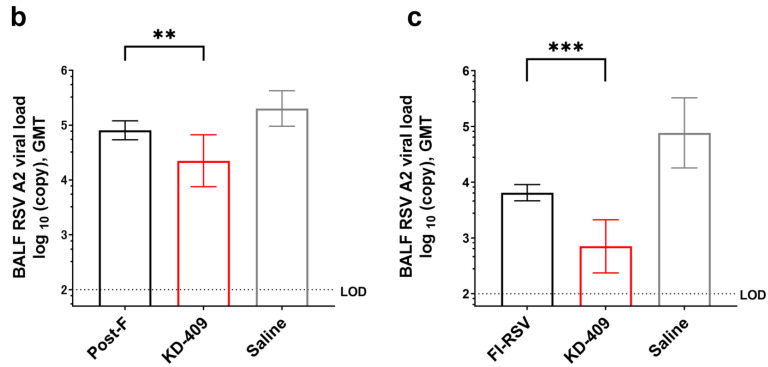
Evaluation of protection against infection by active immunization. Mice inoculated twice with each protein antigen and phosphate–alum adjuvant (none) through intramuscular administration were used to evaluate protection against infection. RSV A2 at 1.0 × 10^5^ PFU was inoculated intranasally, and the number of virus copies in bronchoalveolar lavage fluid (BALF) was measured 3 days after the virus challenge. Error bars indicate the 95% CI. (**a**) Infection-protective efficacy of pre-F, wild-type (WT) F, mutant F with a glycosylated modification of WT at site Ⅳ, and KD-409 (5 µg/dose antigen without Adju-Phos, n = 16). Statistical analysis was performed with the Kruskal–Wallis test and Dunn’s multiple comparison test (** *p* < 0.01, *** *p* < 0.001, **** *p* < 0.0001). (**b**) Comparison of infection-protective efficacy between post-F and KD-409 (15 µg/dose antigen without Adju-Phos, n = 8). Statistical analysis was performed with the two-tailed Mann–Whitney U test (** *p* < 0.01, *** *p* < 0.001). (**c**) Comparison of infection-protective efficacy between formalin-inactivated RSV (FI-RSV) and KD-409 (5 µg/dose antigen with Adju-Phos, n = 7–8). Statistical analysis is the same as in (**b**). LOD, limit of detection.

To assess the protective potential of KD-409 against infection, we conducted an RSV A2 challenge test after two active immunizations with pre-F, post-F, FI-RSV, F glycosylated site IV, and KD-409 in a mouse model. Analysis of the viral copy number in BALF 3 days after infection showed that KD-409 without adjuvant significantly suppressed infection compared to the saline control, demonstrating a level of protection similar to that of RSV pre-F (Figure 3a). Furthermore, KD-409 with aluminum phosphate showed significantly greater protection than classical antigens, such as RSV post-F and FI-RSV (Figure 3b,c). Dose–response analysis in the active immunity mouse model indicated that KD-409 with aluminum phosphate at doses of 15 μg and 0.005 μg significantly outperformed post-F in protecting against infection (Figure S3).

Interestingly, the protective effect in the active immunity mouse model of mutant F glycosylated site IV, which did not contain G_162–171_, was comparable to that of WT F, suggesting that the effect of the inserted G_162–171_, but not site IV glycosylation, improved the ability to protect against infection (Figure 3a). These results indicate that KD-409 immunization surpassed WT F, post-F, and FI-RSV immunization, mainly owing to the effect of inserted G_162–171_, and showed a level of protection similar to that of pre-F.

### 3.4. Protective Efficacy of KD-409 against Infection (Passive Immunity)

**Figure 4:**
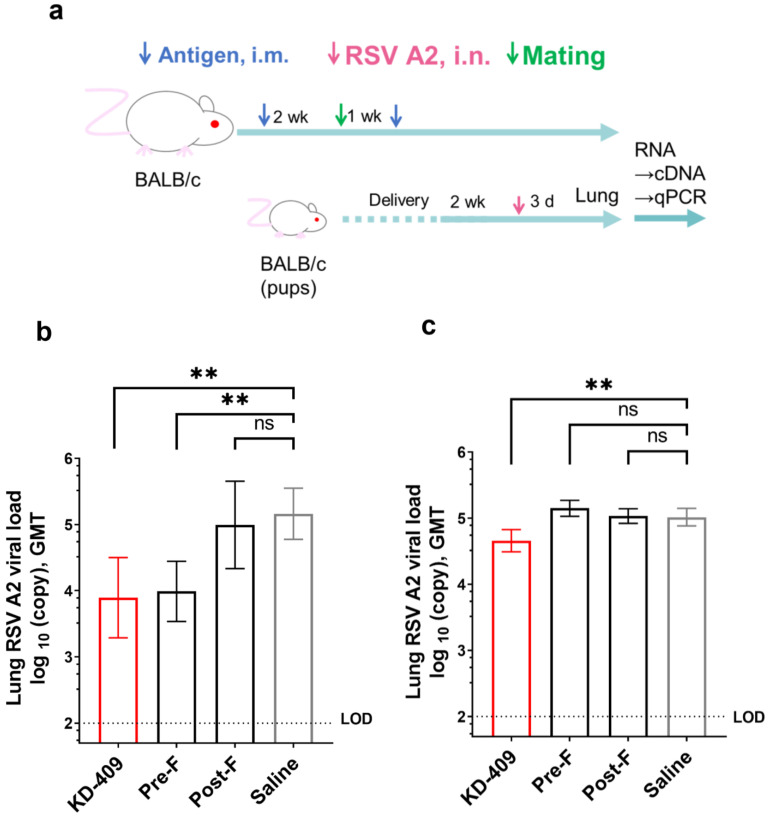
Evaluation of protection against infection through passive immunization. Evaluation of protection against infection using pups born from mothers inoculated twice with each protein and phosphate–alum adjuvant through intramuscular administration. Pups were inoculated intranasally (i.n.) with RSV (A2) at 1.0 × 10^5^ PFU. Virus copy numbers in lung tissue were measured 3 days after the virus challenge. (**a**) Overview of the animal study design. (**b**) Lung viral loads/virus copy numbers were measured using quantitative polymerase chain reaction (qPCR) (15 µg/dose antigen with Adju-Phos, n = 16). (**c**) Lung viral loads/virus copy numbers were measured using qPCR (0.5 ng/dose antigen with Adju-Phos, n = 16). Statistical analysis was performed with the Kruskal–Wallis test and Dunn’s multiple comparison test (^ns^ *p* ≥ 0.05, ** *p* < 0.01). Error bars are the 95% CI. i.m., intramuscular; LOD, limit of detection.

RSV accounts for 2.0% of deaths in children aged 0–60 months and 3.6% of deaths in children aged 28 days to 6 months [3]. The highest rate of hospitalization due to RSV infection occurs in the first 6 months of life, with a particular peak between 2 and 4 months of age [2]. Vaccine efficacy may not be sufficient for the active immunization of newborns to prevent severe diseases, making passive immunization a potential strategy to prevent RSV infection/severe diseases in newborns through the transfer of antibodies after maternal vaccination. Methods using animal models of guinea pigs and monkeys have been used to evaluate passive immunization/pregnant inoculation [36,37]. To evaluate protection against infection in a simplified mouse pregnancy model, we used a mouse model in which IgG was transferred to pups via breast milk and blood (Figure 4a).

In this model, IgG against RSV F was transferred to the pups, and dose dependence was confirmed (Figure S4). In this model, we assessed protection against infection at two different doses of KD-409, 15 μg and 0.0005 µg/dose, representing maximum-efficacy and minimum-efficacy doses, respectively. Analysis of viral copy numbers in lung tissue from pups 3 days after infection showed that at the maximum effective dose, the protective efficacy of KD-409 was comparable to that of pre-F and superior to that of post-F (Figure 4b). At the minimum effective dose, KD-409 also showed protective efficacy in the passive immunization mouse model at a 0.0005 µg/dose. In contrast, neither pre-F nor post-F showed any protective effect against infection at this dose (Figure 4c). At a 0.00005 µg/dose, KD-409, pre-F, and post-F showed no protective effect against infection. In conclusion, the protective efficacy of KD-409 was comparable to that of pre-F and superior to that of post-F at the maximum dose, and KD-409 was more effective than pre-F at the minimum dose.

## 4. Discussion

In this study, we successfully developed a vaccine against RSV infection named KD-409, which is a chimeric protein combining a highly conserved region of the RSV G protein with RSV F. KD-409 demonstrated greater efficacy in protecting against infection compared to the conventional RSV vaccine candidates FI-RSV and post-F. Our efficacy evaluation revealed promising results, with KD-409 demonstrating greater effectiveness than the leading vaccine candidate, pre-F, at the minimum effective dose, positioning it as a potential candidate for an RSV vaccine.

Recent studies have shown that RSV F site IV is exposed on both pre-F and post-F surfaces and that the neutralizing capacity of antibodies to site IV is approximately 1/10 that of antibodies to site Φ [38,39,40,41]. Therefore, the neutralizing capacity may be reduced if anti-site IV antibodies are not induced or if the induced antibody balance, such as sites Φ, Ⅰ, Ⅱ, and/or Ⅲ, is altered by site-IV-modified antigen vaccination. The reduced reactivity of KD-409 with anti-site IV antibodies can be attributed to the introduction of sugar chains into site IV (Figure S2). F-glycosylated site IV, a glycosylation of site IV of the F protein without G_162–171_, showed the same level of infection protection as WT F (Figure 3a), suggesting that at least the efficacy of the WT F protein antigen is not changed by glycosylation of site IV. However, determining the effect of site IV glycosylation of FG chimeras on the infection protection ability was difficult because animal studies could not be conducted owing to the low protein expression of an FG chimera without glycosylation of site IV. Future studies will be required to determine whether this modification results in the induction of antibodies to other sites, including anti-site IV antibodies.

It has been reported that antigen size and shape can influence immunity [42,43]. Indeed, KD-409 is not simply an aggregate but a self-assembly with a characteristic rosette particle structure similar to the structure of post-F, resulting in a different IgG2a/IgG1 ratio than that of pre-F. Our findings suggest that antigen size or shape affects the immune system. Notably, pre-fusion virus-like particle (pre-F VLP) and post-F VLP immunizations have been reported to induce Th1-based immunity [44]. As expected, the anti-G antibody titer after KD-409 inoculation was lower than the anti-F antibody titer owing to the short G sequence. However, the infection protection ability of KD-409 was equal to or greater than that of pre-F. If the contribution of antibodies to the G_162–171_ sequence present at low levels is not significant, then the increase in size due to protein self-assembly may have contributed to the infection protection effect, resulting in Th1-based immune induction similar to those of the VLPs described above. On the other hand, KD-409 has a significantly greater ability to protect against infection than post-F, which forms a rosette-like structure, and it is controversial the extent to which low levels of anti-G antibodies affect its efficacy. This study did not go as far as to investigate the predominant isotype of post-F, and it is not clear what the effects of immunity are on T cell subpopulations (including memory) and NK cells. It will be important to determine whether there is a difference in immune induction with the rosette-like structure or the trimer of KD-409 or whether it is the shape rather than the size that matters.

The neutralizing activity of anti-RSV G antibodies cannot be confirmed if strain-derived HeLa cells are used [13]. The reason for this may be that CX3CR1 on the cell surface, which is the primary receptor for the RSV G protein, is shed, leaving only HS, with its limited receptor function, as the primary point of interaction between the virus and the host cell. In fact, in a test system using human airway epithelial cells (hAECs) expressing CX3CR1, the anti-G-CCD antibody showed neutralizing activity comparable to that of the anti-F antibody [45]. Neutralizing antibody titers have also been reported to be altered by the presence or absence of complements [46]. In this study, we used the Hep-2 cell line derived via HeLa contamination with or without complement. Although anti-G antibodies were induced through KD-409 immunization, neutralizing antibody titers were lower than those of pre-F in the absence of complement and higher than those of pre-F in the presence of complement. The presence of complement is more typical of the in vivo environment, and IgG2a with its complement-binding properties may have been influenced the results of neutralizing antibody titers. These results suggest that KD-409 outperforms pre-F in terms of neutralizing antibody titers. However, given the influence of cell lines and the presence or absence of complement in the neutralizing antibody titer assay system and the fact that mouse IgG1 inhibits complex formation with IgG2a, IgG2b, IgG3, and C1q [47], a more accurate assessment of efficacy may require the use of animal models rather than neutralizing antibody titers alone.

It is important to consider that the mouse pregnancy model used in this study differs from animal models that more strictly mimic human antibody transfer, as IgG is transferred through breast milk instead of the placenta. Further evaluation in animal models more closely resembling human antibody transfer is required. In a previously reported evaluation using a guinea pig model, antibody transfer was confirmed through immunization with RSV F as the antigen [48]. Our data showed that the minimum effective dose of KD-409 outperformed pre-F in a mouse passive immunization model; however, given the actual mode of antibody transfer in humans, we confirmed IgG transfer in a guinea pig model. In addition, the cotton rat model has traditionally been used to evaluate the efficacy of RSV vaccines [36]. Cotton rats are approximately 100-fold more permissive and immunologically reactive than BALB/c mice, with 10-fold higher serum antibody titers [49,50]. The present study showed that the efficacy of KD-409 was comparable to that of pre-F in a mouse model of active immunity, but it will be important to evaluate these findings in cotton rats and other mouse models.

G_162–171_ is not the CX3C motif itself, which is considered an adhesion and aggravation factor for host cells, but rather a region adjacent to the CX3C motif in the 3D structure [29]. Including G_162–171_ in chimeric proteins of the G protein ectodomain and F has been shown to be more potent than FI-RSV, but it is unclear whether these proteins are more potent than pre-F. In addition, there are significant concerns regarding symptom exacerbation due to the inclusion of the CX3C motif [22,23]. KD-409 does not contain the CX3C motif found in conventional FG chimeric proteins, such as BBG2Na, and only G_162–171_, among the highly conserved regions of G, is incorporated into F. KD-409 eliminates the potential for symptom exacerbation caused by the CX3C motif while still inducing antibodies against epitopes around CX3C. Additionally, FG chimeric proteins are expected to possess medicinal properties that are more potent than those of pre-F considering their minimum effective dose (Figure 4c). This study only reported results with a focus on the efficacy of FG chimeric proteins. Considering the exacerbation of symptoms in an FI-RSV clinical trial in the 1960s [20], adverse reactions with BBG2Na, including the CX3C motif and BB carrier protein as antigens [21], and the exacerbation of symptoms with pre-F and post-F low-dose immunization [18], including preterm birth among pregnant individuals following RSVPreF vaccination [51], further studies are required to verify whether KD-409 can effectively prevent adverse events, including exacerbations. In the near future, we will report the results of our safety evaluation study of KD-409 for use in infants of all ages and children.

In conclusion, we successfully developed a novel FG chimeric vaccine antigen, KD-409, and demonstrated its superiority over conventional FI-RSV and post-F antigens. KD-409 outperformed the promising pre-F antigen in a passive immunity mouse model, indicating its potential as a next-generation RSV vaccine for neonates and infants.

## Data Availability

The datasets presented in this article are not readily available because the data are part of an ongoing study. Requests to access the datasets should be directed to the corresponding author.

## Supplementary Materials

The following supporting information can be downloaded at: https://www.mdpi.com/article/10.3390/vaccines12070753/s1, Figure S1: Coomassie-Brilliant-Blue-stained SDS-PAGE for each antigen; Figure S2: Reactivity of each antigen with anti-respiratory syncytial virus (RSV) F antibodies determined according to absorbance; Figure S3: Dose–response evaluation of protection against infection; Figure S4: Anti-RSV F antibody titers of 2wk pups born from mothers inoculated twice with KD-409 and Adju-Phos by intramuscular administration.
