# Supplementary material for: KD-409, a Respiratory Syncytial Virus FG Chimeric Protein without the CX3C Chemokine Motif, Is an Efficient Respiratory Syncytial Virus Vaccine Preparation for Passive and Active Immunization in Mice
Source: Vaccines (Basel). 2024 Jul 8;12(7):753. doi: 10.3390/vaccines12070753 (PMC11281633; doi:10.3390/vaccines12070753)
Supplement: Supplementary file 1 [file vaccines-12-00753-s001.zip › vaccines-3068493-supplementary.pptx]

## Slide 1
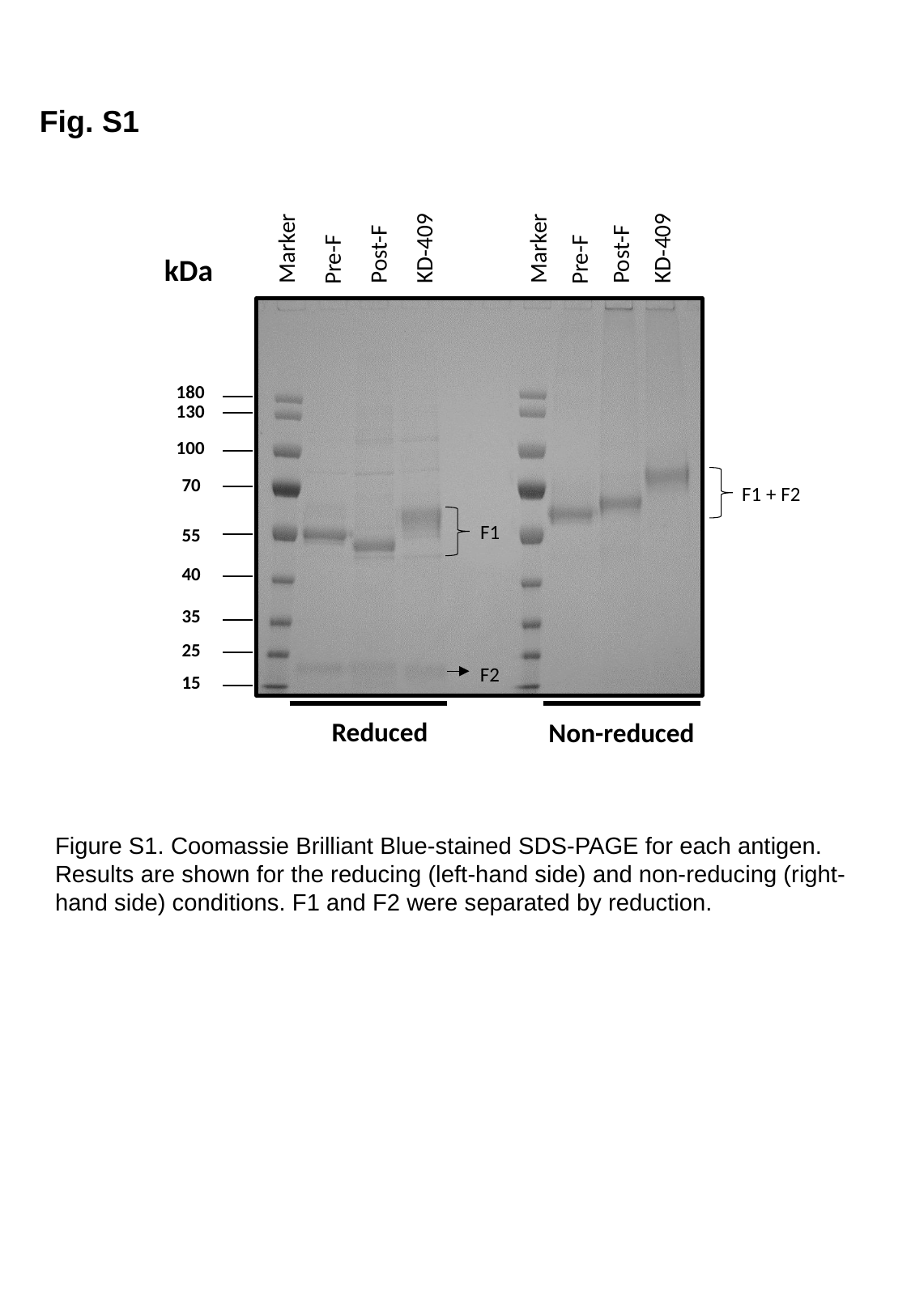

Fig. S1
KD-409
KD-409
Marker
Marker
Post-F
Post-F
Pre-F
Pre-F
kDa
180
130
100
70
55
40
35
25
15
Reduced
Non-reduced
F1 + F2
F1
F2
Figure S1. Coomassie Brilliant Blue-stained SDS-PAGE for each antigen. Results are shown for the reducing (left-hand side) and non-reducing (right-hand side) conditions. F1 and F2 were separated by reduction.

## Slide 2
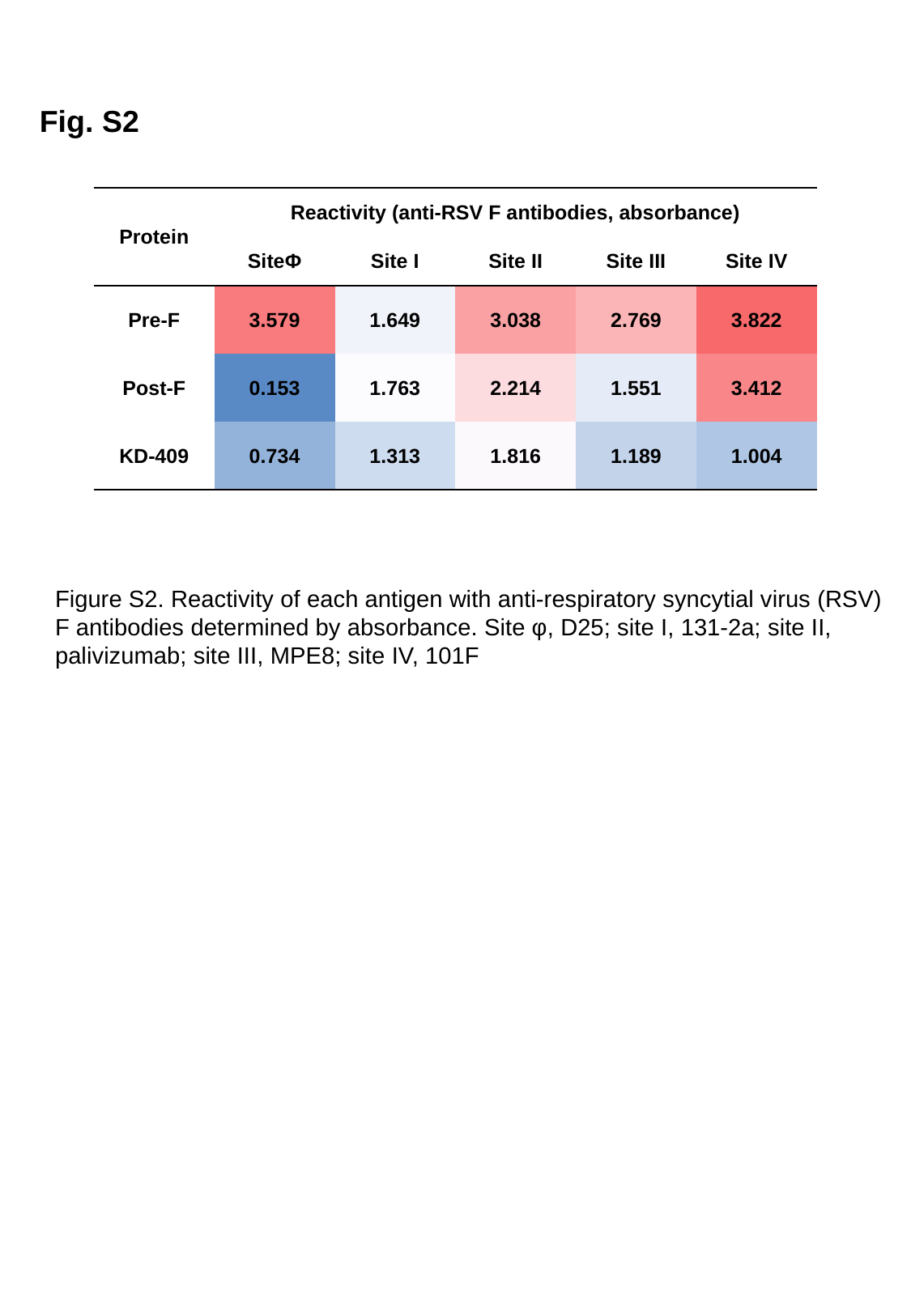

Fig. S2
| | | | | | | | |
| --- | --- | --- | --- | --- | --- | --- | --- |
| | Protein | Reactivity (anti-RSV F antibodies, absorbance) | | | | | |
| | | SiteΦ | Site I | Site II | Site III | Site IV | |
| | Pre-F | 3.579 | 1.649 | 3.038 | 2.769 | 3.822 | |
| | Post-F | 0.153 | 1.763 | 2.214 | 1.551 | 3.412 | |
| | KD-409 | 0.734 | 1.313 | 1.816 | 1.189 | 1.004 | |
| | | | | | | | |
Figure S2. Reactivity of each antigen with anti-respiratory syncytial virus (RSV) F antibodies determined by absorbance. Site φ, D25; site I, 131-2a; site II, palivizumab; site III, MPE8; site IV, 101F

## Slide 3
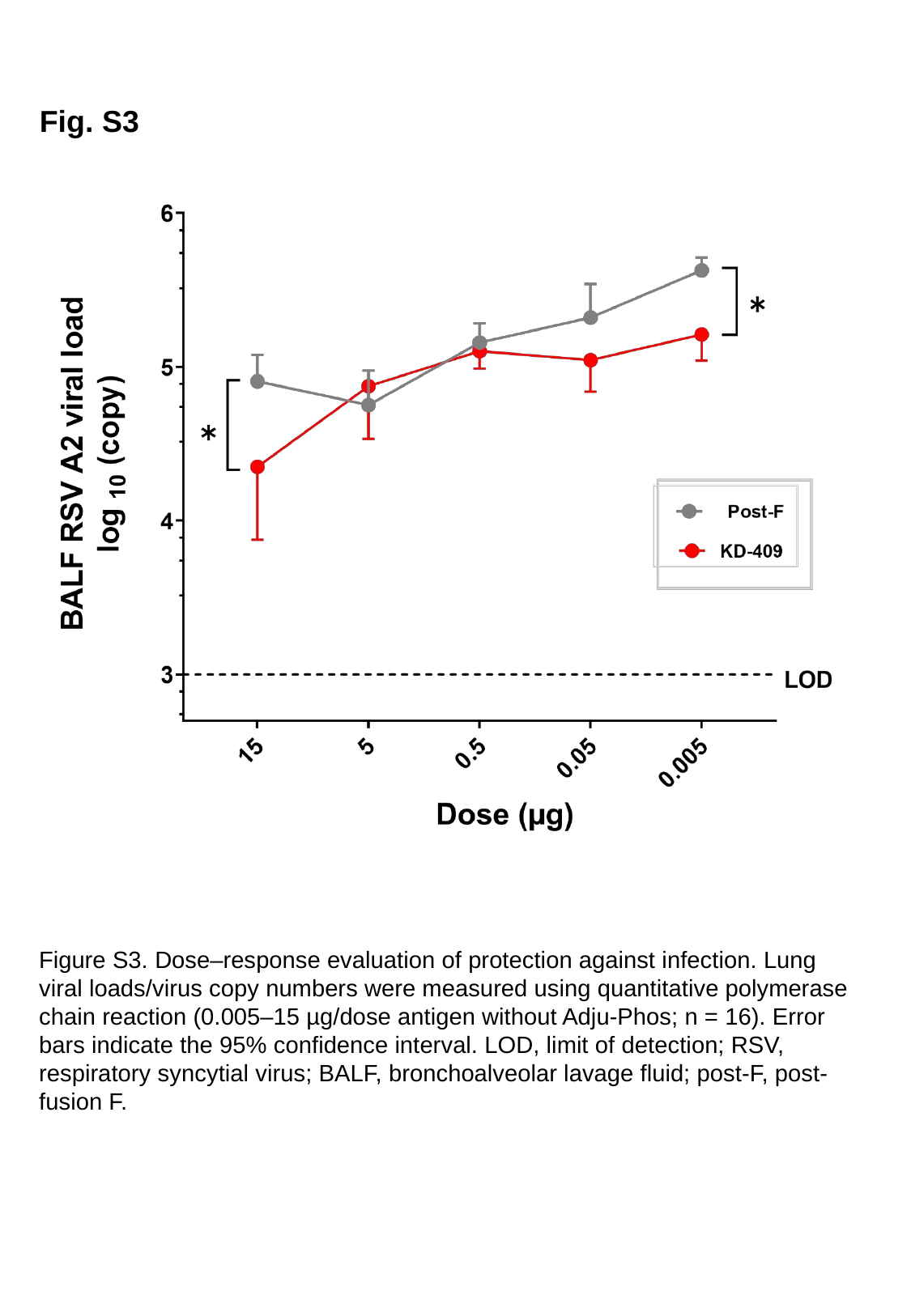

Fig. S3
Figure S3. Dose–response evaluation of protection against infection. Lung viral loads/virus copy numbers were measured using quantitative polymerase chain reaction (0.005–15 µg/dose antigen without Adju-Phos; n = 16). Error bars indicate the 95% confidence interval. LOD, limit of detection; RSV, respiratory syncytial virus; BALF, bronchoalveolar lavage fluid; post-F, post-fusion F.

## Slide 4
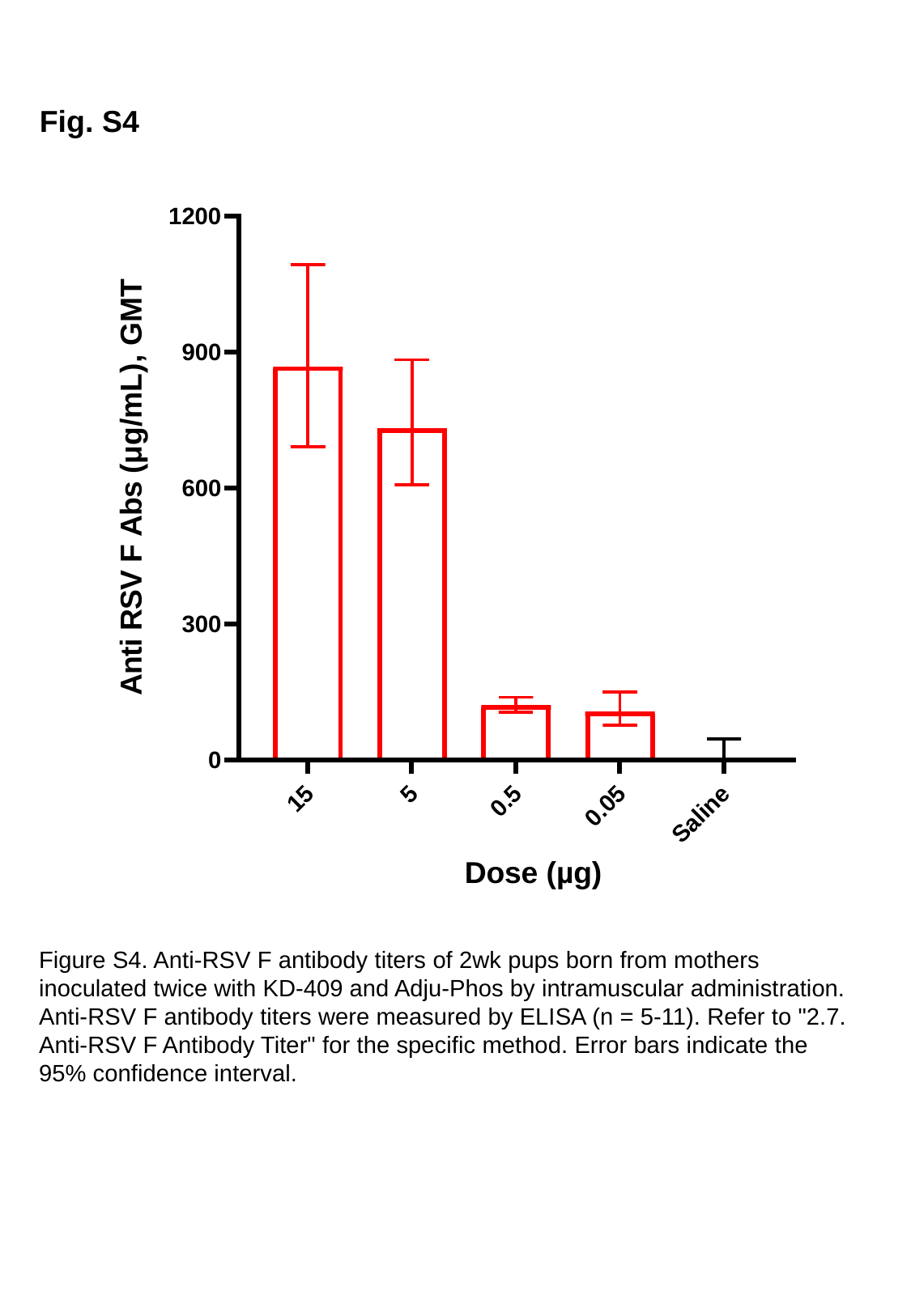

Fig. S4
Figure S4. Anti-RSV F antibody titers of 2wk pups born from mothers inoculated twice with KD-409 and Adju-Phos by intramuscular administration. Anti-RSV F antibody titers were measured by ELISA (n = 5-11). Refer to "2.7. Anti-RSV F Antibody Titer" for the specific method. Error bars indicate the 95% confidence interval.
